# Frailty-Driven Prediction of Inpatient Obstructive Sleep Apnea and Related Sleep Disorder Diagnoses Using Explainable AI

**DOI:** 10.3390/biomedicines14061304

**Published:** 2026-06-08

**Authors:** Assiya Boltaboyeva, Bibars Amangeldy, Zhanel Baigarayeva, Baglan Imanbek, Nurdaulet Tasmurzayev, Adilet Kakharov, Sultan Tuleukhanov, Zhanar Omirbekova, Balzhan Makhatova

**Affiliations:** 1 AlfaCenter (Al-Farabi AI Center), Farabi University, Almaty 050040, Kazakhstan; 2 LLP “Kazakhstan R&D Solutions”, Almaty 050056, Kazakhstan; 3 Faculty of Biology and Biotechnology, Farabi University, Almaty 050040, Kazakhstan; 4 Institute of Automation and Information Technologies, Satbayev University, Almaty 050056, Kazakhstan; 5 Faculty of Pharmacy, Asfendiyarov Kazakh National Medical University, Almaty 050012, Kazakhstan; 6 National Academy of Science of Kazakhstan under the President of the Republic of Kazakhstan, Almaty 050010, Kazakhstan

**Keywords:** sleep disorders, obstructive sleep apnea, insomnia, frailty, hospital frailty risk score, MIMIC-IV, XGBoost, gradient boosting, machine learning, clinical decision support, electronic health records, SHAP, inpatient prediction, sleep-disordered breathing

## Abstract

**Background/Objectives:** Obstructive sleep apnea (OSA) and related sleep disorders affect a substantial proportion of hospitalized patients, with an estimated 48% pooled prevalence of undiagnosed OSA in cardiac inpatients and up to 80% of moderate-to-severe community OSA cases carrying no formal diagnosis at the time of hospital admission. In parallel, frailty—a state of heightened physiological vulnerability arising from cumulative multi-system biological decline—is present in 40–80% of inpatients and shares deep, bidirectional neurobiological pathways with sleep-disordered breathing through circadian dysregulation, intermittent hypoxia, hypothalamic–pituitary–adrenal axis activation, and chronic low-grade inflammation. Despite this convergence, no prior study has integrated validated, administratively computable frailty phenotyping with a machine learning framework specifically designed to predict inpatient sleep disorder diagnosis—and OSA in particular—at the point of hospital admission. The present study addresses this gap by developing an admission-time, explainable machine learning framework for the prediction of inpatient sleep disorder diagnoses (ICD-10 G47.x, encompassing OSA G47.3, insomnia G47.0, hypersomnia, and circadian rhythm disorders) and of insomnia specifically (ICD-10 G47.00). **Methods:** We developed and evaluated a suite of five binary classification models—XGBoost, Random Forest, LightGBM, CatBoost, and Decision Tree—using 9682 balanced hospitalization episodes from the MIMIC-IV (version 2.2) database. The predictor set comprised 23 admission-time structured features across three domains: (i) frailty and comorbidity burden, including the Hospital Frailty Risk Score (HFRS) derived from ICD-10 codes, the Elixhauser comorbidity index, prior admission history, and six binary disease flags (obesity, hypertension, type 2 diabetes, heart failure, COPD, and depression/anxiety); (ii) physiological and laboratory biomarkers from the first 24 h of care, including minimum SpO_2_, heart rate variability, hemoglobin, creatinine, albumin, and arterial blood gas parameters; and (iii) sociodemographic and administrative variables encompassing age, sex, ethnicity, insurance type, and admission acuity. Model performance was assessed through five-fold stratified cross-validation and bootstrap confidence intervals (*n* = 1000 iterations), with predictor importance quantified using SHapley Additive exPlanations (SHAP). **Results:** XGBoost achieved the strongest aggregate performance across all evaluation metrics, attaining an area under the receiver operating characteristic curve (AUC) of 0.871 (95% CI: 0.856–0.887), accuracy of 79.6%, F1-score of 0.820, and sensitivity of 94.9%, correctly identifying 903 of 952 true positive cases in the held-out test set; all gradient boosting frameworks substantially outperformed the Decision Tree baseline (AUC 0.836). SHAP analysis identified the HFRS and Elixhauser index as the two dominant predictors, followed by depression/anxiety, obesity, hypertension, and minimum SpO_2_—a hierarchy that recapitulates the canonical clinical phenotype of obstructive sleep apnea in frail inpatients rather than that of primary insomnia, indicating that the model is preferentially capturing the OSA–frailty axis within the broader G47.x outcome. The predicted probability outputs were well-calibrated across all risk deciles. **Conclusions:** Frailty-derived features, in combination with admission-time clinical and physiological data, can predict inpatient sleep disorder diagnoses—predominantly OSA—with high sensitivity and well-calibrated risk estimates. The deployable, interpretable nature of the XGBoost model makes it directly suitable for integration into clinical decision support systems, offering a screening tool that requires no dedicated instrumentation beyond routine admission data. By flagging high-risk patients at the moment of admission, the framework provides a concrete mechanism for accelerating referral for definitive diagnostic confirmation (overnight oximetry, polysomnography) and earlier initiation of CPAP and related therapies, with direct implications for reducing the persistent diagnostic gap, perioperative risk, and preventable adverse outcomes in frail hospitalized populations.

## 1. Introduction

The accelerating aging of the global population has fundamentally transformed the epidemiology of hospital admissions. This demographic shift places a new class of patients at the center of acute care medicine: those who are not simply sick, but frail. Frailty is broadly defined as a state of heightened physiological vulnerability arising from cumulative and multi-system biological decline. It affects approximately 17% of community-dwelling adults aged 60 and above. However, its prevalence in the acute inpatient setting surges to between 40% and 80%, depending on the specific population studied [1,2]. This disproportionate representation is not coincidental. Frail individuals are more susceptible to the physiological insults of acute illness. They are more likely to experience functional deterioration during hospitalization and are significantly more prone to readmission and mortality compared to non-frail patients with equivalent comorbidity profiles [3]. What makes frailty particularly challenging is that it cannot be reduced to any single disease. Instead, it is an emergent property of cumulative biological erosion across the musculoskeletal, cardiovascular, immune, and neuroendocrine systems. This erosion manifests as weakness, exhaustion, weight loss, slow gait, and diminished physiological resilience [1]. Understanding and predicting adverse outcomes in frail hospitalized patients, therefore, demands a multidimensional clinical lens. Such an approach must integrate structured comorbidity data, laboratory biomarkers, vital signs, socioeconomic context, and longitudinal hospitalization history into a coherent predictive framework.

Operationalizing frailty at scale in hospital systems has long been constrained by the practical limitations of physical assessment tools. Instruments such as the Fried Phenotype Criteria or the Clinical Frailty Scale require direct patient evaluation. This process is resource-intensive and difficult to standardize across large and heterogeneous inpatient cohorts. A pivotal advance came with the development of the Hospital Frailty Risk Score (HFRS) by Gilbert et al., which is a validated tool derived exclusively from ICD-10 diagnostic codes captured in routine hospital administrative data [4]. By assigning weighted scores to 109 frailty-associated diagnoses and stratifying patients into low, intermediate, and high frailty risk categories, the HFRS enabled population-scale frailty identification from existing data without creating any additional clinical burden. Validated across national cohorts in the United Kingdom, Canada, Switzerland, Germany, and France, the HFRS has consistently demonstrated predictive validity for prolonged hospitalization, 30-day readmission, and mortality [5,6]. Complementing this frailty proxy is the Elixhauser Comorbidity Index. This index enumerates up to 38 ICD-10-coded chronic conditions into a weighted summary score. It provides an independently validated measure of disease burden that substantially enriches risk stratification when integrated into machine learning pipelines [7,8]. Together, these two administratively computable constructs offer a robust and clinically meaningful feature infrastructure for predictive modeling in large electronic health record (EHR) databases such as MIMIC-IV.

Against this backdrop of frailty and multimorbidity, one adverse clinical phenomenon has remained conspicuously under-addressed in the inpatient literature, namely, sleep disorders. Sleep is not a passive physiological state. Rather, it is an active biological process essential for immune regulation, neuroendocrine homeostasis, cellular repair, and cognitive consolidation. Its disruption in the hospital environment is both pervasive and clinically consequential. Patients admitted to acute care wards are exposed to a convergence of sleep-disruptive forces. These include continuous ambient lighting, frequent nursing assessments, monitoring alarms, procedural interruptions, pain, dyspnea, and the psychological burden of acute illness. Such factors systematically fragment sleep architecture, reduce slow-wave and rapid eye movement sleep duration, and dysregulate the circadian phase [9]. Despite this near-universal exposure, sleep disorders remain profoundly underdiagnosed in hospital settings. Among cardiac inpatients, undiagnosed obstructive sleep apnea (OSA) has been documented at a pooled prevalence of approximately 48%. Furthermore, population-level estimates suggest that up to 80% of individuals with moderate-to-severe OSA in the community carry no formal diagnosis at the time of hospitalization [9,10]. The pathophysiology of OSA is particularly relevant in the context of frailty-laden inpatient populations. Upper airway collapse during sleep is exacerbated by reduced pharyngeal muscle tone—a direct consequence of sarcopenia—and by increased central adiposity, which reduces chest wall compliance and elevates the apnea-hypopnea index. Frailty-associated comorbidities independently amplify OSA severity: heart failure promotes both obstructive and central sleep-disordered breathing through elevated pulmonary capillary wedge pressure and augmented loop gain [11]; COPD compounds nocturnal hypoxia through impaired hypercapnic ventilatory response; and type 2 diabetes contributes through autonomic neuropathy and nocturnal oxygen desaturation. Left unrecognized and untreated, OSA in hospitalized patients is independently associated with increased risks of postoperative cardiovascular complications, arrhythmia, delirium, and prolonged mechanical ventilation [11,12]. In psychiatric inpatient cohorts, chronic sleep disorders, which are typically identified via ICD-10 codes, hypnotic prescriptions, or clinical documentation, were present in over 81% of admissions. These disorders were independently associated with higher rehospitalization rates, greater use of physical restraint, and a broader burden of comorbid conditions compared to patients without documented sleep pathology [13]. These figures collectively underscore a stark diagnostic reality. Within the inpatient setting, sleep disorders are far more common than they are currently recognized, documented, or treated.

Insomnia, classified under ICD-10 code G47.00, occupies a central position within this diagnostic gap. It is defined by a persistent difficulty initiating or maintaining sleep despite adequate opportunity. This condition is accompanied by daytime dysfunction, including fatigue, cognitive impairment, and mood dysregulation, and it exerts direct adverse effects on hospital recovery trajectories [14]. Its identification in clinical practice depends heavily on patient self-reports and clinician recognition. However, neither of these factors is systematically encoded in structured electronic health record (EHR) fields. The ICD-10 G47.x family thus represents only a fraction of true inpatient sleep pathology. Furthermore, the likelihood of receiving a formal diagnosis during hospitalization is shaped not only by symptom severity but also by factors such as age, insurance type, admission acuity, and provider practice patterns. These factors are themselves structured and predictable from admission-time data [14]. This reality creates a compelling case for a data-driven approach. If the clinical and administrative profiles of patients at high risk of an inpatient sleep disorder diagnosis can be learned from historical EHR data, then this knowledge can be deployed prospectively to flag at-risk patients at the moment of admission before any formal diagnosis has been assigned. This would enable targeted clinical attention and earlier intervention.

The frailty–sleep connection is a particularly compelling predictive target because of the depth and bidirectionality of their biological relationship. The association between frailty and sleep disturbance is not merely a coincidental clustering of comorbidities; rather, it reflects shared and mutually reinforcing neurobiological mechanisms. In a cohort of 540 older hospitalized patients, Shen et al. demonstrated that poor sleep quality was independently associated with an elevated frailty risk. The odds of frailty increased substantially when sleep disturbance co-occurred with depressive symptoms or chronic pain [15]. A meta-analysis of observational studies confirmed that insomnia carries a significant pooled association with frailty in older populations. This association is mediated through shared phenotypic features, including fatigue, physical inactivity, impaired gait speed, and weight loss [16]. Importantly, Lu et al. applied two-sample Mendelian randomization to establish causal directionality using genetic variants as instrumental variables. They identified a statistically robust causal pathway from sleep disturbances to increased frailty risk. This suggests that sleep dysfunction is not merely a downstream consequence of aging and multimorbidity, but rather an upstream driver that accelerates frailty progression [17]. This causal insight elevates the clinical importance of identifying sleep disorders early in hospitalized patients.

At the neurophysiological level, circadian dysregulation serves as a pivotal mechanistic bridge between frailty and sleep pathology. Cai et al. conducted a landmark prospective cohort study published in Nature Communications, which demonstrated that fragmented rest–activity circadian rhythms are robustly and independently associated with incident frailty. These rhythms were quantified objectively via wrist actigraphy over 16 years of longitudinal follow-up, independent of age, sex, and baseline disease burden [18]. This circadian frailty nexus is mediated, in part, through neuroendocrine dysregulation. Sleep fragmentation rapidly activates the hypothalamic–pituitary–adrenal (HPA) axis, elevating serum glucocorticoid concentrations within one hour of sleep disruption. This subsequently triggers the systemic release of pro-inflammatory cytokines, including interleukin-6 (IL-6) and tumor necrosis factor-alpha (TNF-α) [19]. This inflammatory cascade constitutes the molecular substrate of the “inflammaging” phenotype-a chronic, low-grade, sterile inflammation that is a cardinal feature of biological aging and the frailty syndrome. Persistently elevated inflammatory markers impair muscle protein synthesis, accelerate sarcopenia, and compromise immune surveillance. Simultaneously, they disrupt sleep architecture through altered serotonergic and adenosinergic regulatory systems, creating a self-reinforcing biological loop that deepens both frailty severity and sleep pathology over time [18,19]. Specific comorbidities that define high-frailty hospitalization profiles amplify this dynamic. Heart failure drives obstructive and central sleep-disordered breathing through elevated pulmonary capillary wedge pressure; COPD disrupts sleep continuity via nocturnal oxygen desaturation and heightened sympathetic tone; type 2 diabetes contributes through nocturia and peripheral neuropathic pain; and depression and anxiety, which are prominent features of frailty cluster analyses, are among the most consistently identified clinical predictors of insomnia across both community and inpatient populations [9,15,16].

Despite this robust theoretical and empirical foundation, the clinical literature reveals a notable absence of predictive models specifically designed to identify sleep disorder diagnoses in hospitalized patients at the time of admission. Prior machine learning studies on sleep disorders have relied almost exclusively on community-based survey datasets, polysomnographic laboratory cohorts, or patient-reported symptom questionnaires. Such data sources are rarely available at scale in acute care environments. For example, Huang et al. developed an XGBoost-based model for insomnia risk prediction using data from the National Health and Nutrition Examination Survey (NHANES), achieving an AUROC of 0.87. Nevertheless, this model was neither trained nor validated on electronic health record (EHR) data from hospitalized populations, and it did not incorporate frailty-specific biomarkers [20]. Similarly, Kim et al. extended this methodology to multi-center polysomnographic cohorts, utilizing XGBoost with SHAP-based feature selection to classify obstructive sleep apnea (OSA), comorbid insomnia, and sleep apnea with strong predictive performance [21]. Because polysomnography is not routinely performed during standard hospital admissions, the clinical applicability of such models to the inpatient EHR context remains constrained. In the context of the MIMIC-IV database, gradient boosting and ensemble methods have consistently demonstrated strong performance across numerous clinical prediction tasks, including mortality, ICU readmission, acute kidney injury, and acute respiratory distress syndrome. Algorithms such as XGBoost, LightGBM, and CatBoost have achieved AUROCs between 0.82 and 0.92 across various inpatient cohorts [22,23,24]. To date, however, no published study has integrated HFRS-derived frailty scoring, Elixhauser comorbidity indices, prior admission history, vital sign parameters, frailty-sensitive laboratory biomarkers, and sociodemographic variables into a unified machine learning framework specifically targeting sleep disorder diagnosis. This omission is particularly consequential given that OSA—the single most prevalent and clinically actionable condition within the G47.x spectrum—remains undertreated even when diagnosed [25]. An admission-time predictive model that identifies frailty signatures associated with G47.x diagnoses, therefore, functions, in clinical practice, primarily as an OSA pre-screening tool, channeling high-risk patients toward formal polysomnographic evaluation or empirical CPAP titration during the inpatient stay.

The present study addresses this gap by developing and evaluating a comprehensive machine learning framework to predict inpatient diagnoses of any sleep disorder (ICD-10: G47.x) and insomnia specifically (ICD-10: G47.00). The model relies exclusively on structured clinical data available at the time of admission from the MIMIC-IV database. Our feature set is grounded in the frailty phenotyping paradigm and incorporates the HFRS calculated from admission ICD-10 codes, the Elixhauser comorbidity index, the cumulative number of prior hospital admissions, discharge disposition from the most recent encounter, and patient age as a validated frailty proxy. These baseline features are supplemented with targeted comorbidity indicators for obesity (E66.x), hypertension (I10), type 2 diabetes (E11.x), heart failure (I50.x), chronic obstructive pulmonary disease (J44.x), and depression or anxiety (F32.x, F41.x). The dataset also includes frailty-sensitive laboratory markers (hemoglobin, creatinine, albumin), physiological parameters collected within the first 24 h (BMI, peripheral oxygen saturation (SpO_2_), and heart rate variability), arterial blood gas measurements when available, and sociodemographic variables such as sex, ethnicity, insurance type as a proxy for socioeconomic status, and admission acuity. We trained and compared five classification algorithms (XGBoost, Random Forest, LightGBM, CatBoost, and Decision Tree) within a rigorous cross-validated experimental framework. Model interpretability was assessed using SHAP analysis to identify the most clinically influential predictors. This study introduces the first predictive model specifically targeting inpatient sleep disorder diagnosis and provides the first systematic benchmark of gradient boosting methods for this clinical outcome. It also represents the initial integration of validated frailty phenotyping tools with sleep medicine prediction in an acute care setting. This convergence offers direct implications for the development of clinical decision support systems and the design of hospital-based sleep screening programs.

The methodological framework of this study specifically prioritizes Explainable AI (XAI) to ensure clinical transparency and build trust among healthcare providers. While deep learning and transfer learning architectures are currently highly popular, their primary advantages lie in processing unstructured data such as medical imaging or clinical notes. For structured, tabular electronic health record data, tree-based machine learning ensembles not only offer competitive or superior predictive performance but also allow for precise, feature-level interpretability. Because clinical implementation requires a clear understanding of how a model arrives at its diagnostic predictions, we focused exclusively on algorithms that inherently support exact explainability methods like SHAP, deliberately avoiding the “black box” nature of deep neural networks.

This study presents several novel contributions to the field of clinical informatics and sleep medicine. To our knowledge, this is the first study to integrate administratively computable frailty phenotyping, specifically the Hospital Frailty Risk Score and Elixhauser Comorbidity Index, with machine learning to predict inpatient sleep disorder diagnoses at the time of admission. By relying solely on standard, admission-time electronic health record data, the proposed models offer a scalable screening tool that does not require immediate, costly, and resource-intensive polysomnography. Furthermore, we provide a benchmark of gradient boosting frameworks, including XGBoost, LightGBM, and CatBoost, on the MIMIC-IV database for this specific clinical target, enhanced by SHAP-based explainable AI to ensure clinical transparency.

## 2. Materials and Methods

This investigation leveraged de-identified electronic health record data from the Medical Information Mart for Intensive Care IV (MIMIC-IV, version 2.2), a publicly available critical care repository encompassing over 300,000 inpatient admissions at the Beth Israel Deaconess Medical Center (Boston, MA, USA) from 2008 to 2022. Access was secured under a PhysioNet data use agreement, and institutional ethical review was waived due to the fully de-identified nature of the dataset. The analytic cohort was constructed by integrating core relational tables (admissions, patients, diagnoses icd, chart events, and lab events), linking each unique hospitalization episode through HADM_ID and STAY_ID identifiers. To preserve prospective clinical utility, the entire predictor set was restricted to variables available at admission or derivable from the first 24 h of care, ensuring that any deployed model could generate risk stratification at the point of entry before sleep pathology is formally documented. Prior to balancing, the original dataset exhibited a significant class imbalance, comprising 60,525 episodes without a sleep disorder diagnosis (majority class) and 4841 episodes with a diagnosis (minority class). To mitigate modeling bias while maintaining ecological validity, random downsampling of the majority class was applied without replacement (random_state = 42), yielding a balanced cohort of 9682 hospitalization episodes (4841 positive and 4841 negative instances). Before finalizing this strategy, we empirically evaluated alternative class-imbalance techniques, including the Synthetic Minority Over-sampling Technique (SMOTE) and class-weighted loss functions. However, applying SMOTE to our complex, multidimensional clinical EHR data, which contains mixed categorical and continuous variables, introduced significant synthetic noise, resulting in notably weaker predictive performance. Consequently, random downsampling was selected as the optimal strategy, ensuring the models learn exclusively from real, unadulterated patient records. This balanced dataset was subsequently partitioned into training and validation sets using an 80/20 stratified random split (random_state = 42). Stratification was applied based on the primary outcome to ensure consistent class prevalence across both cohorts. Critically, to prevent data leakage, patient-level partitioning was enforced: unique patient identifiers were used to guarantee that all hospitalization records for any single patient were confined exclusively to either the training or the validation set, with no overlap between cohorts. This rigorous approach ensures that the model generalizes to new patients rather than merely memorizing individual subject patterns from the training data. Two binary classification targets were defined independently: the primary outcome captured any sleep disorder diagnosis within the ICD-10 G47.x spectrum—with obstructive sleep apnea (G47.3x) constituting the numerically dominant subgroup, followed by insomnia (G47.0x), hypersomnia, and circadian rhythm disorders. Given the epidemiological predominance of OSA within hospitalized populations and its established association with frailty-related comorbidities, the G47.x primary outcome was specifically designed to function as an OSA-inclusive screening target. The secondary outcome isolated insomnia specifically (ICD-10: G47.00). Critically, both endpoints were restricted to diagnoses assigned during the index admission only, ensuring the modeling task predicts incident diagnostic recognition rather than historical condition retrieval.

**Figure 1:**
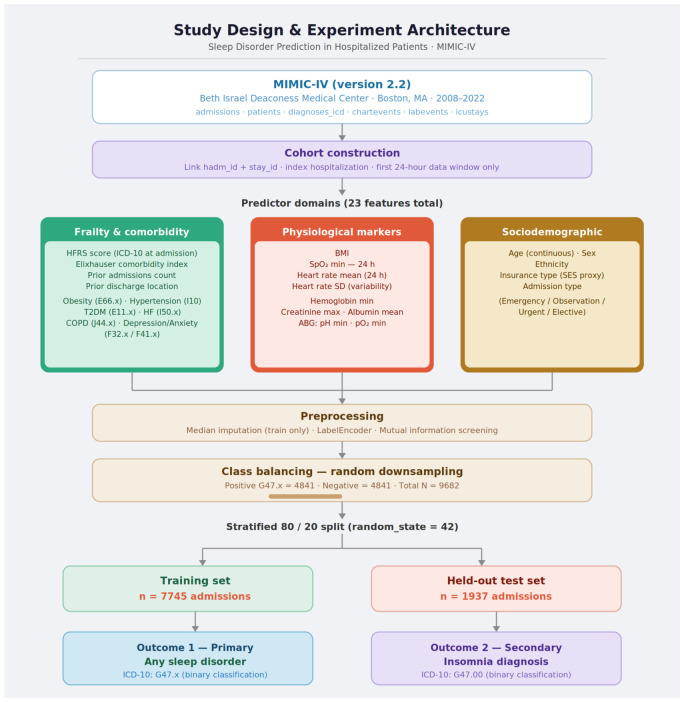
Study Design and Experiment Architecture.

The predictor architecture comprised 23 admission-time features organized across three clinically coherent domains, as delineated in Figure 1. The first domain quantified frailty and multimorbidity burden through the Hospital Frailty Risk Score (HFRS), the Elixhauser comorbidity index, cumulative prior admission counts, discharge disposition from the most recent prior encounter, and six binary ICD-10 flags for obesity (E66.x), hypertension (I10), type 2 diabetes (E11.x), heart failure (I50.x), COPD (J44.x), and depression/anxiety (F32.x/F41.x). The second domain integrated physiological and laboratory biomarkers that were captured within the first 24 h, including body mass index (BMI), minimum peripheral oxygen saturation (SpO_2_), mean heart rate, heart rate variability (standard deviation), minimum hemoglobin, maximum creatinine, mean albumin, and arterial blood gas parameters (minimum pH and pO_2_) when clinically available. The third domain encompassed sociodemographic and administrative determinants, specifically continuous age, biological sex, self-reported ethnicity, insurance type as a validated socioeconomic status proxy, and admission acuity (emergency, observation, urgent, or elective). Together, these structured variables form a comprehensive, admission-available feature set designed to capture the multidimensional pathophysiology linking systemic inflammation, physiological vulnerability, and inpatient sleep pathology, while maintaining strict adherence to the prospective prediction paradigm outlined in the study architecture. All data preprocessing, model training, and evaluation procedures were implemented in Python version 3.13. The experimental pipeline utilized several key open-source libraries, including scikit-learn version 1.6.1 for general machine learning utilities and evaluation metrics, xgboost version 3.1.3, lightgbm version 4.3.0, and catboost version 1.2.8 for the gradient boosting algorithms. Interpretability analysis was conducted using the shap library version 0.49.1. Hyperparameter tuning was performed using RandomizedSearchCV to optimize the models efficiently while preventing overfitting. Computational experiments were executed on a workstation equipped with an AMD Ryzen 9 9950X processor (Advanced Micro Devices, Inc., Santa Clara, CA, USA), 64 GB of RAM, and an RTX5090 GPU (Nvidia Corporation, Santa Clara, CA, USA), running on a Windows 11 operating system.

### 2.1. Data Preprocessing

Data extraction and preliminary processing were performed using DuckDB, which enabled efficient aggregation of raw clinical data directly from compressed Parquet files. The cohort was filtered to exclude pediatric patients (under 18 years of age) and was restricted to each patient’s first intensive care unit (ICU) admission. This approach ensured the statistical independence of observations and prevented data leakage that could arise from repeated hospitalizations of the same individual.

Feature engineering was subsequently conducted to construct a robust, clinically meaningful predictor set. Comorbidity indices were derived using established weighting systems: the Hospital Frailty Risk Score (HFRS) was calculated by mapping 109 ICD-10 diagnostic codes to their corresponding empirical weight coefficients, and the Elixhauser comorbidity score was computed using the van Walraven weighting system. For physiological and laboratory parameters, temporal feature engineering was applied to capture the most clinically relevant baseline values. Specifically, data were extracted and aggregated within a strictly defined time window ranging from 6 h before to 24 h after ICU admission. This approach ensured that critical tests performed during emergency department care prior to ICU transfer were included in the baseline profile. Because this 23-feature set was carefully selected a priori based on the clinical frailty paradigm and given the inherent feature selection capabilities of tree-based gradient boosting algorithms, no additional automated dimensionality reduction was required prior to modeling.

For the secondary outcome (insomnia), the target variable included not only the primary ICD-10 code G47.00 but also the related codes G47.01 and G47.09. This broader definition accounted for variations in clinical documentation and improved case capture. Finally, the dataset was prepared for modeling using standard machine learning preprocessing techniques. Categorical variables were encoded numerically, and missing values in continuous clinical and laboratory variables were addressed using statistical imputation. These steps ensured the balanced dataset was optimized for model training.

Specifically, we observed that while demographic variables and calculated scores had complete data, certain clinical biomarkers exhibited substantial missingness (e.g., Arterial blood gases: 36–38%, Heart Rate, SpO2, Creatinine, Hemoglobin: <3%, Age, Comorbidities, calculated scores (HFRS, Elixhauser): 0%). We explicitly acknowledge that in EHR data, such missingness is clinically non-random (Missing Not at Random, MNAR), as these tests are ordered selectively based on patient acuity. To fully leverage the native missing-value handling capabilities of gradient boosting architectures, missing values were left un-imputed (retained as NaN) for the XGBoost, LightGBM, and CatBoost models. Median imputation was employed strictly to standardize the input space for the baseline Decision Tree algorithm. By relying directly on the missing data distribution for the primary ensemble models, we mitigated the potential bias that explicit imputation would otherwise introduce.

To ensure the integrity of the predictive framework and prevent data leakage, all feature engineering, normalization, and statistical imputation procedures were fitted exclusively on the training subset. These fitted parameters (e.g., means and standard deviations for continuous variables, or mode values for imputation) were subsequently applied to the validation subset. This strict isolation ensures that the validation results reflect the model’s performance on unseen clinical scenarios, maintaining the validity of the prospective prediction paradigm.

### 2.2. ML Models

A binary classification approach was used to predict sleep disorder onset in critically ill patients. The analytical framework included both simple interpretable models and advanced ensemble methods. A Decision Tree algorithm served as the baseline to establish the basic predictive capacity of the feature set. The primary focus was on ensemble architectures, specifically Random Forest and three gradient boosting algorithms (XGBoost, LightGBM, and CatBoost). Random Forest uses a bagging strategy to reduce prediction variance, while the gradient boosting methods build sequential decision trees to improve accuracy. These gradient boosting algorithms were selected because they perform well with heterogeneous tabular data, handle correlated clinical predictors effectively, and natively manage missing values. These characteristics are essential for analyzing real-world electronic health records.

Model training and validation procedures were designed to minimize overfitting. The balanced dataset was split into training and hold-out test sets using an 80:20 ratio. Stratification was applied to maintain consistent class distribution across both sets. During model development, generalization performance was estimated using stratified 5-fold cross-validation on the training cohort. Hyperparameter optimization was performed using RandomizedSearchCV. Key parameters included maximum tree depth, learning rate, L1/L2 regularization, and subsampling fractions (see Appendix A, Table A1). This approach balanced computational efficiency with thorough parameter tuning. The reliability of the results was assessed by calculating 95% confidence intervals for all key metrics using bootstrap resampling with 1000 iterations on the hold-out test set.

Because clinical decision support systems require transparent and interpretable models, we applied Explainable Artificial Intelligence (XAI) methods to address the typical opacity of ensemble algorithms. The SHapley Additive exPlanations (SHAP) framework was used to interpret the best-performing model. SHAP values are derived from cooperative game theory and provide a consistent method for explaining model predictions. SHAP analysis provided both global and local interpretability. Globally, it ranked clinical and laboratory features by their overall contribution to the model. Locally, it identified how each predictor increased or decreased the probability of an insomnia diagnosis for individual patients.

Statistical tests were conducted to compare model performance. A bootstrap hypothesis test compared the Area Under the Receiver Operating Characteristic Curve (AUC) of the best-performing model (XGBoost) against the other classifiers. For threshold-dependent metrics such as accuracy, McNemar’s test evaluated differences in prediction contingency tables. Statistical significance was defined as a two-sided *p*-value < 0.05.

## 3. Results

The predictive efficacy of the developed machine learning algorithms was evaluated on an independent hold-out test set, representing 20% of the initial balanced dataset (n = 1937 admissions). To comprehensively assess the discriminatory capacity and reliability of the classifiers, a robust suite of metrics was utilized, including the Area Under the Receiver Operating Characteristic Curve (ROC AUC), Accuracy, Precision (Positive Predictive Value), Sensitivity (Recall), and the F1-score. The performance outcomes of all baseline and ensemble models are summarized in Table 1.

An analysis of the results demonstrates a substantial advantage of ensemble machine learning methods over baseline algorithms. The Decision Tree, serving as the baseline model, exhibited moderate generalization capabilities with an ROC AUC of 0.8357. Despite its interpretability, the single tree was prone to overfitting and failed to fully capture the complex non-linear interactions among clinical predictors.

The transition to Random Forest and gradient boosting architectures (XGBoost, LightGBM, CatBoost) yielded statistically significant improvements (*p* < 0.001) across all key metrics compared to the baseline. The Random Forest algorithm achieved the highest absolute sensitivity (Recall ≈ 0.9600); however, this was accompanied by a decrease in Precision, indicating the model’s tendency toward overdiagnosis (an increased rate of false positives). The gradient boosting models, LightGBM and CatBoost, demonstrated high and stable efficacy, achieving ROC AUCs of 0.8683 and 0.8701, respectively, with balanced F1-scores (≈0.82).

Crucially, formal statistical testing revealed that the performance differences among the top four ensemble models (XGBoost, Random Forest, LightGBM, and CatBoost) were not statistically significant (all pairwise *p*-values > 0.05 for both McNemar’s test of accuracy and the Bootstrap test of AUC). Although XGBoost achieved the highest nominal ROC AUC of 0.8708 and an overall Accuracy of 79.50%, these algorithms demonstrated comparably robust predictive capacities. Nevertheless, based on its optimal empirical equilibrium between minimizing false-negative outcomes (Sensitivity of 0.9500) and maintaining overall prognostic accuracy, XGBoost was selected as the representative final model for further graphical deconstruction and clinical interpretation.

Regarding computational time, all models demonstrated exceptionally high efficiency, making them highly suitable for real-time clinical deployment. The training times on the analytic dataset were under one second for all models, ranging from 0.04 s for the baseline Decision Tree to 0.828 s for the Random Forest (LightGBM: 0.05 s; XGBoost: 0.191 s; CatBoost: 0.394 s). Most importantly, the inference time required to generate a prediction for a single patient episode was practically instantaneous across all algorithms: 0.0005 ms (Decision Tree), 0.0010 ms (LightGBM), 0.0015 ms (CatBoost), 0.0021 ms (XGBoost), and 0.0103 ms (Random Forest). This microsecond-level latency ensures that our predictive framework can be seamlessly integrated into hospital Electronic Health Record (EHR) systems without causing computational bottlenecks.

**Figure 2:**
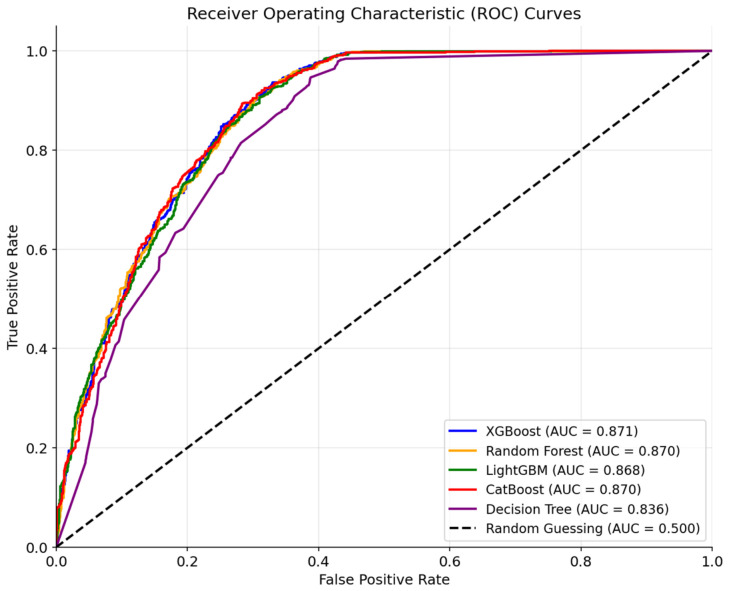
Comparison of Receiver Operating Characteristic (ROC) curves across all evaluated predictive models.

As illustrated in Figure 2, the ensemble machine learning models demonstrate superior discriminatory power compared to the baseline Decision Tree. All four ensemble algorithms (XGBoost, Random Forest, LightGBM, and CatBoost) achieved comparably high performance, with Area Under the ROC Curve (AUC) values ranging from 0.868 to 0.871, significantly outperforming the Decision Tree (AUC = 0.836).

While the XGBoost model achieved the highest nominal AUC of 0.871, the proximity of the ROC curves for all ensemble methods suggests that they share a robust and stable predictive capacity. Given its optimal equilibrium between sensitivity and specificity, the XGBoost model was selected for further clinical interpretation. For this model, an optimal classification threshold of 0.52 was identified, ensuring a balanced trade-off between the True Positive Rate (Sensitivity) and the False Positive Rate (1-Specificity) for clinical deployment.

**Figure 3:**
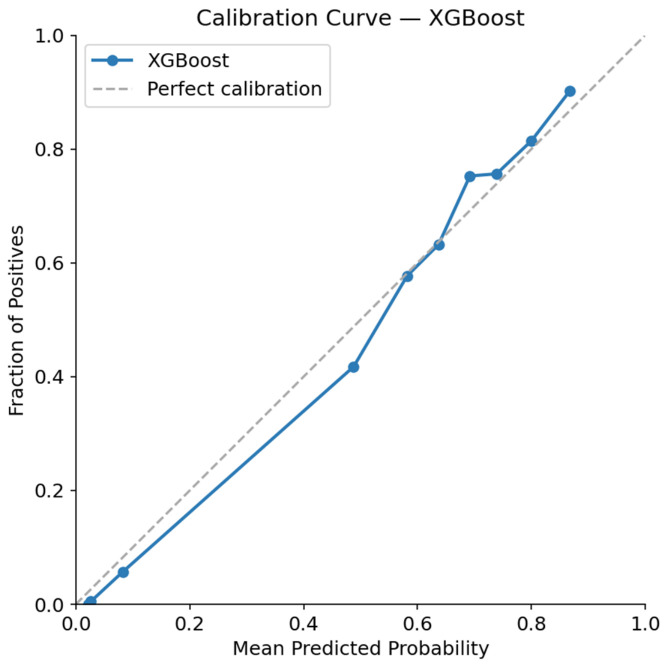
Calibration Curve of the XGBoost model.

To assess how accurately the model’s predicted probabilities reflect the true underlying frequency of the event, a calibration curve was constructed (Figure 3). The plot demonstrates a high degree of concordance: the empirical data points closely align with the perfect calibration diagonal. This indicates that if the model predicts a 70% risk of developing insomnia, approximately 70% of the patients in that quantile will genuinely have the diagnosis. Such calibration reliability is paramount for integrating the algorithm into Clinical Decision Support Systems (CDSS).

**Figure 4:**
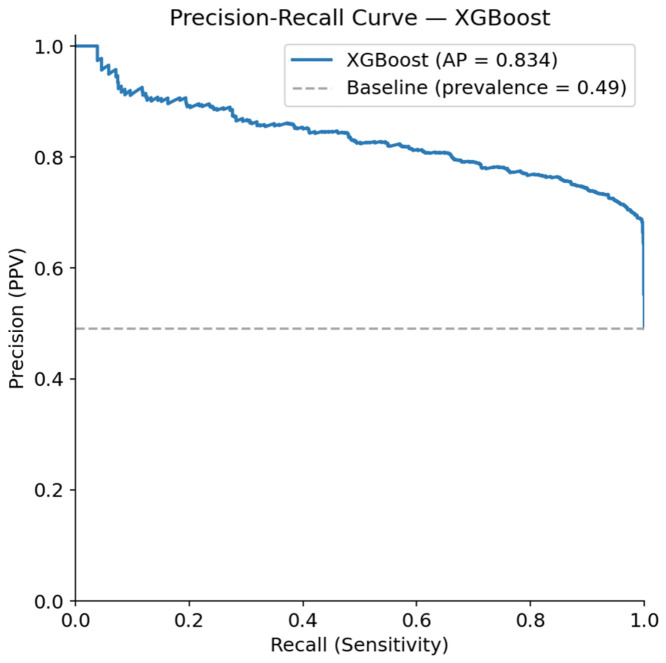
Precision–Recall Curve of the XGBoost model.

Considering that classes can be imbalanced in real-world clinical practice, the Precision–Recall curve was additionally analyzed (Figure 4). The Average Precision (AP) score was 0.834. The graph confirms the algorithm’s robustness: XGBoost successfully maintains high predictive precision (PPV) even when operating at extremely high recall thresholds (Recall > 0.8).

**Figure 5:**
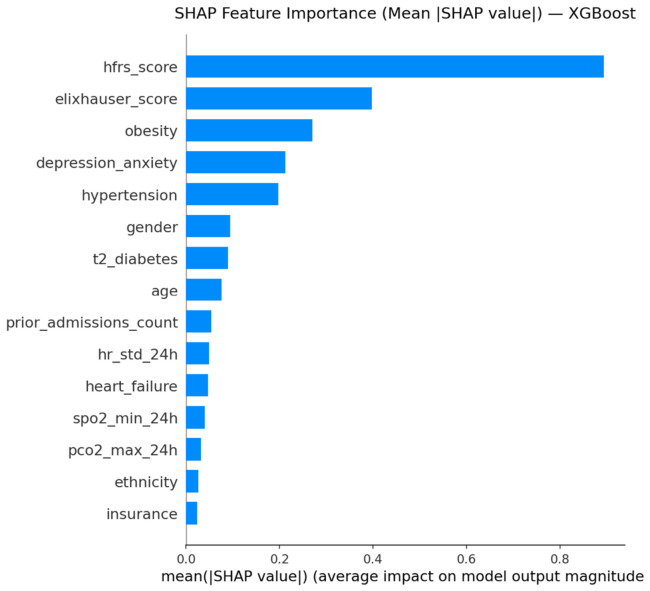
SHAP Global Feature Importance.

To overcome the “black-box” dilemma, the SHAP methodology was applied. The global importance plot (Figure 5) ranks the predictors based on the mean absolute value of their contribution to the model’s output. A fundamental finding was that integral metrics of premorbid vulnerability—the Hospital Frailty Risk Score (HFRS) and the Elixhauser Comorbidity Index (elixhauser score)—are the dominant predictors, significantly outweighing isolated demographic or vital signs in importance. 

**Figure 6:**
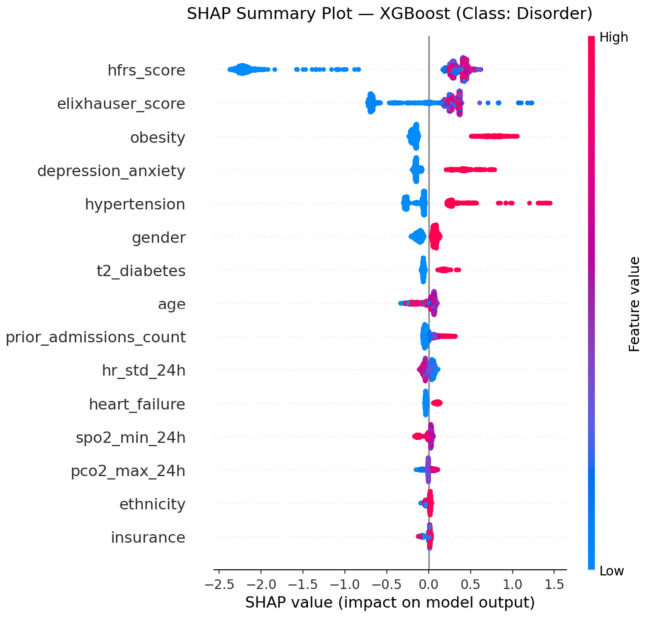
SHAP Summary Plot: local impact and directionality of predictors.

The detailed directionality of these impacts is presented in the SHAP Summary Plot (Figure 6). The analysis reveals a strict biological gradient: extremely high values of the frailty and comorbidity indices (marked in red) generate elongated positive SHAP vectors, exponentially increasing the risk of sleep disorders. The presence of specific comorbidities, such as obesity, hypertension, and depression/anxiety, similarly shifts the prediction conclusively toward pathology. Concurrently, among the acute phase parameters (first 24 h), low minimum oxygen saturation values (SpO_2_ min 24 h, blue dots) are associated with elevated risk, reflecting the pathogenetic link between acute hypoxia and the disintegration of sleep architecture. Importantly, this predictor pattern maps coherently onto the clinical phenotype of OSA: the co-occurrence of elevated HFRS, obesity, depression, and reduced SpO_2_ constitutes precisely the frailty-metabolic-hypoxic cluster that is most strongly associated with sleep-disordered breathing severity in hospitalized adults. The model’s dominant predictors thus reflect not only general sleep pathology risk, but specifically the pathophysiological determinants of obstructive sleep apnea within this population. 

**Figure 7:**
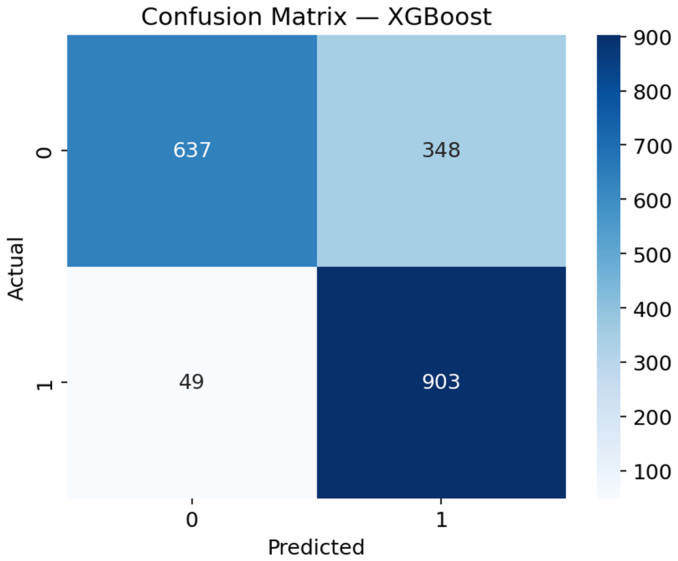
Confusion Matrix of the XGBoost classification model.

The ultimate assessment of clinical applicability is reflected in the confusion matrix (Figure 7). Within the test cohort of 1937 patients, the algorithm successfully identified 903 out of 952 actual cases of sleep disorders, committing only 49 false-negative errors. This ensures a clinically highly acceptable sensitivity of 94.8%. The number of false-positive alarms was 348, which represents an acceptable trade-off for a screening tool in critical care, where the primary objective is to avoid overlooking a vulnerable patient.

## 4. Discussion

To our knowledge, this study presents the first machine learning framework specifically designed to predict inpatient sleep disorder diagnoses using only structured data available at hospital admission from the MIMIC-IV database. The framework targets both the broad sleep disorder spectrum (ICD-10: G47.x) and insomnia specifically (ICD-10: G47.00). Across all five evaluated algorithms, gradient boosting methods substantially outperformed the Decision Tree baseline. XGBoost emerged as the top-performing model, achieving an ROC AUC of 0.871, an overall accuracy of 79.6%, and a sensitivity of 94.8% on the held-out test set.

These results hold clear clinical relevance. A sensitivity approaching 95% means that fewer than 5 in 100 patients who would ultimately receive a sleep disorder diagnosis during hospitalization are missed by the model at admission. This performance threshold supports the framework’s viability as a prospective clinical screening tool. The broader results show consistent performance across the ensemble methods: XGBoost (AUC 0.871), CatBoost (AUC 0.870), LightGBM (AUC 0.868), and Random Forest (AUC 0.870). The main distinction among these models lies in their balance between sensitivity and precision rather than in overall discriminatory capacity.

The superiority of gradient boosting methods over a single Decision Tree aligns with established evidence on machine learning for structured clinical data. In a large-scale benchmark study spanning 45 tabular datasets, Grinsztajn et al. demonstrated that tree-based ensemble methods, particularly XGBoost, LightGBM, and CatBoost, consistently outperform both simpler classifiers and deep learning architectures when handling heterogeneous tabular data with mixed feature types [26]. This condition matches the multimodal EHR-derived feature set used in our study. When directly comparing the three gradient boosting frameworks on medical classification tasks, XGBoost has shown the most robust accuracy and generalization across diverse datasets. LightGBM offers superior computational efficiency for large datasets, while CatBoost performs best when categorical features are numerous and high cardinality [27]. In our setting, where categorical features such as insurance type, admission type, ethnicity, and discharge disposition were relatively limited, and the dataset was moderate in size, XGBoost’s strength in regularization and variance control likely contributed to its consistent advantage. This produced the best calibrated probability outputs for integration into clinical decision support systems.

Regarding the methodological choices, we deliberately bypassed deep learning and transfer learning architectures in favor of advanced gradient boosting frameworks. Although deep learning excels in unstructured data domains, recent empirical benchmarks demonstrate that tree-based algorithms consistently outperform deep neural networks on structured tabular datasets, which are characterized by heterogeneous features, unscaled values, and irregular decision boundaries. More importantly, the central conceptual pillar of our research is Explainable AI. Deep learning models function essentially as opaque black boxes; while model-agnostic explanation tools like LIME or LIME-based SHAP can be applied to neural networks, they provide only approximate explanations and are computationally prohibitive for large tabular datasets. In contrast, tree-based models leverage TreeSHAP, which calculates exact Shapley values efficiently. This allows for reliable, highly detailed interpretations of how specific clinical variables-such as the Hospital Frailty Risk Score-drive individual predictions. This exact level of transparency, which deep learning cannot currently guarantee, is an absolute prerequisite for integrating predictive models into real-world clinical decision support systems.

SHAP analysis revealed that the Hospital Frailty Risk Score (HFRS) and the Elixhauser Comorbidity Index were the strongest predictors of inpatient sleep disorders. These two indices had the highest mean absolute SHAP values, substantially exceeding the predictive contribution of any demographic, vital sign, or laboratory variable. This result aligns with the study design and current clinical understanding. The HFRS captures cumulative biological vulnerability across 109 ICD-10-coded conditions. Patients with high frailty scores typically present with multiple comorbidities, including depression, chronic pain, cardiovascular disease, and respiratory impairment. These conditions are well-established clinical drivers of sleep pathology [16,18]. The Elixhauser index further strengthens this signal by measuring the overall chronic disease burden associated with frailty. Together, these two administratively derived indices act as effective proxies for the complex biological risk factors that predispose hospitalized patients to sleep disorders. This finding supports the study’s theoretical framework and confirms that integrating frailty phenotyping into predictive modeling is a clinically sound approach.

Among specific comorbidities, depression and anxiety (F32.x/F41.x), obesity (E66.x), and hypertension (I10) had the strongest positive influence on model predictions. This pattern aligns with established clinical literature. The relationship between depression and insomnia is well-documented in sleep medicine. A meta-analysis by Baglioni et al. showed that insomnia doubles the risk of subsequent depression, while pre-existing depression significantly increases the likelihood of developing and maintaining insomnia. This creates a reciprocal clinical relationship that worsens both conditions [28]. Machine learning studies focusing on sleep and mental health comorbidities have similarly identified depression and anxiety as top predictive features in both community and hospital cohorts [26,27,28,29]. The high predictive value of obesity in our model reflects its established role as a major modifiable risk factor for obstructive sleep apnea (OSA). Excess adipose tissue in the upper airway reduces lumen size and decreases chest wall compliance. This increases the apnea-hypopnea index and contributes to both OSA and comorbid insomnia [30]. Kurnool et al. further described how obesity, sleep apnea, and diabetes interact as a reinforcing metabolic cycle. Disrupted sleep worsens insulin resistance and appetite regulation, which promotes further weight gain and exacerbates sleep-disordered breathing [31].

The top predictors identified by SHAP—frailty burden, obesity, depression, and minimum SpO_2_—constitute a clinically coherent OSA risk signature. This convergence has direct therapeutic implications. Hospitalized patients who carry this profile are precisely those most likely to benefit from in-hospital CPAP initiation, positional therapy, or perioperative airway monitoring. Evidence from randomized controlled trials and systematic reviews supports the use of CPAP in hospitalized OSA patients to reduce postoperative cardiovascular events, decrease sympathetic nervous system activation, improve oxygen desaturation indices, and attenuate the HPA-axis inflammatory cascade that is central to both OSA severity and frailty progression [12,32,33]. An explainable AI model that surfaces this profile at admission, therefore, aligns directly with the therapeutic optimization goals of the present Special Issue: it converts an administratively invisible risk into an actionable clinical prompt that can trigger appropriate workup and treatment before discharge.

Minimum SpO_2_ recorded during the first 24 h of admission also emerged as a significant physiological predictor. Oxygen desaturation during sleep is both a result of sleep-disordered breathing and a direct contributor to sleep fragmentation. Intermittent hypoxia triggers sympathetic nervous system activation, increases cortisol levels, and disrupts the adenosine-driven sleep pressure needed for sustained sleep [34]. In a cohort of patients with OSA, Kumagai et al. found that lower minimum SpO_2_ during sleep was significantly associated with greater disease severity and more negative mood states upon waking. This supports the use of SpO_2_ as a practical, continuously monitored indicator of hypoxic sleep burden [35]. Because SpO_2_ is routinely collected through standard hospital monitoring, it can be incorporated into the prediction framework without requiring specialized sleep assessments or additional equipment.

Our results compare favorably with previous machine learning studies on sleep disorder prediction while offering distinct clinical advantages. Huang et al. developed an XGBoost model using the NHANES community survey dataset and achieved an AUC of 0.87 for predicting insomnia, with depression, sex, and obesity as the top features [20]. Although their discrimination performance is similar to ours, their model was not validated in hospitalized populations, did not include frailty metrics, and relies on self-reported survey data that are not captured during standard hospital admissions. Similarly, Kim et al. reported strong predictive performance using polysomnographic and questionnaire data from multiple centers [21]. However, polysomnography is rarely available during acute hospital admission, which limits the use of such models to specialized sleep clinics. In contrast, our framework relies exclusively on structured clinical and administrative data available at admission. This design enables direct integration into existing hospital electronic health record systems as a real-time clinical decision support tool, without requiring additional patient interviews, specialized equipment, or specialist consultations.

Several limitations of this study warrant explicit acknowledgment. First, the models were trained and validated using a single-center retrospective cohort from the MIMIC-IV database originating from Beth Israel Deaconess Medical Center (BIDMC). Consequently, the generalizability of the trained models to hospitals with different patient demographics, coding practices, or care delivery systems has not been established. Because no external validation on an independent dataset (such as eICU or the UK Biobank) was performed, prospective validation in a multi-center cohort is required before clinical implementation. Second, the target variable of sleep disorders was defined based on ICD-10 diagnostic codes rather than the clinical gold standard of objective polysomnography. This pronounced reliance on administrative coding introduces systematic underdiagnosis bias and label noise: sleep disorders are documented only when clinicians recognize and record them, meaning the negative class likely contains a substantial proportion of patients with undiagnosed sleep pathology-a structural limitation shared by all EHR-based diagnostic prediction studies [14]. Based on external epidemiological estimates, while the true prevalence of sleep disorders in hospitalized adults often approaches 40–50% [10], routine administrative coding typically captures less than 10%. Consequently, a significant proportion, potentially up to 30–40% of our negative class, may represent misclassified latent positives. Crucially, this directional label noise fundamentally alters the construct validity of the classification task by deflating, rather than inflating, our model performance metrics. When the machine learning model correctly identifies the multidimensional physiological and frailty signature of an undiagnosed sleep disorder, it is penalized as a ‘false positive’ against the imperfect administrative ground truth. Therefore, we hypothesize that the reported performance metrics represent a conservative lower bound of the models’ true discriminatory capacity. Third, the statistical imputation methods used to handle missing laboratory and clinical values, while robust, may introduce subtle biases into the predictive models. Fourth, to address the significant original class imbalance (60,525 negative versus 4841 positive episodes), random downsampling was applied to generate a 1:1 balanced cohort. While we empirically evaluated alternative techniques such as the Synthetic Minority Over-sampling Technique (SMOTE) and class-weighted loss functions, applying SMOTE to complex clinical EHR data with mixed categorical and continuous variables introduced synthetic noise and degraded predictive performance. Downsampling was, therefore, selected to ensure the models learned exclusively from real, unadulterated patient records, though this approach inherently discards a large portion of the majority class data. Furthermore, because the models were trained and calibrated on an artificially balanced 50/50 dataset, the raw predicted probabilities do not reflect real-world clinical prevalence. For prospective clinical application, these probability outputs would require posterior recalibration or decision-threshold adjustment to align with the true epidemiological baseline of the deploying institution.

Notwithstanding these limitations, this work makes a substantive contribution to the intersection of frailty research, inpatient sleep medicine, and clinical machine learning. The demonstration that HFRS and Elixhauser-based frailty phenotyping, combined with admission-time clinical and physiological data, can predict inpatient sleep disorder diagnosis with an AUC exceeding 0.87 and a sensitivity approaching 95% opens a concrete pathway toward data-driven, early identification of at-risk patients well before any diagnostic documentation occurs. Future work should pursue external multi-center validation, exploration of temporal feature dynamics across the hospitalization course, and the design of prospective clinical trials evaluating whether model-guided early sleep screening improves patient outcomes, including length of stay, functional recovery, and readmission rates. Integration of this framework into clinical decision support infrastructure, supported by SHAP-based explanations as advocated by Stiglic et al. for interpretable healthcare AI [36], represents a clinically actionable and technically feasible next step toward reducing the substantial and persistent gap between sleep disorder prevalence and diagnosis in the acute inpatient setting.

## 5. Conclusions

This study demonstrates that the inpatient diagnosis of sleep disorders, encompassing both broad spectrum (ICD-10: G47.x) and insomnia specifically (ICD-10: G47.00), can be predicted with high discriminatory performance and near-95% sensitivity. The model relies exclusively on structured data available at hospital admission from the MIMIC-IV electronic health record database. Among the five evaluated classification algorithms, XGBoost achieved the strongest overall performance, with an AUC of 0.871, a sensitivity of 94.9%, and an F1-score of 0.820. All gradient boosting frameworks substantially outperformed the interpretable Decision Tree baseline. Importantly, the well-calibrated probability outputs of the XGBoost model ensure that predicted risk scores accurately reflect true event rates across all risk deciles. This calibration makes the model directly suitable for integration into clinical decision support systems that require transparent and actionable risk estimates for bedside clinicians.

SHAP interpretability analysis yielded a finding of significant theoretical importance. The Hospital Frailty Risk Score and the Elixhauser Comorbidity Index emerged as the two most dominant predictors of inpatient sleep disorder diagnosis, substantially outweighing individual vital sign or demographic variables. This result validates the central hypothesis of the study: frailty, measured through an administratively computable ICD-10-based proxy, captures a multidimensional biological vulnerability that is deeply intertwined with sleep pathology in hospitalized patients, and in addition to these two frailty indices, depression and anxiety, obesity, and minimum SpO_2_ consistently ranked among the most important predictors. This predictor triad—comorbid mood disorder, central adiposity, and nocturnal hypoxemia—maps directly onto the established pathophysiological cascade of obstructive sleep apnea, suggesting that the model’s G47.x predictions are functionally dominated by OSA risk phenotypes. Early identification of this cluster at admission enables clinicians to initiate sleep-disordered breathing workup during the inpatient stay and to consider CPAP therapy or supplemental oxygen as targeted therapeutic interventions, aligning the model’s clinical use case with the emerging evidence base for in-hospital OSA management and therapeutic optimization. Their prominence aligns with established pathophysiological mechanisms linking these conditions to sleep-disordered breathing, insomnia, and nocturnal hypoxemia, reinforcing their value as clinical screening targets.

Several limitations define the current scope of this work. The model was developed and validated using data from a single academic medical center. External validation on independent multi-site cohorts, such as eICU or AmsterdamUMCdb, is necessary to confirm generalizability. Defining the outcome using ICD-10 diagnostic codes introduces the underdiagnosis bias that is common to all EHR-based studies. The true prevalence of sleep pathology in this cohort almost certainly exceeds the documented coding rate. Additionally, the current framework focuses on binary diagnostic prediction and does not account for sleep disorder severity, duration, or response to clinical interventions.

Despite these limitations, this study establishes a reproducible, interpretable, and clinically actionable foundation for electronic health record-driven sleep disorder detection in acute care settings. The next necessary step is a prospective evaluation to determine whether model-guided early identification of at-risk patients improves clinical management, reduces length of stay, or lowers readmission rates. By integrating validated frailty phenotyping with advanced gradient boosting and explainable AI, this framework provides a practical pathway to reduce the persistent gap between the actual prevalence of sleep disorders and their clinical recognition in hospitalized patients.

## Figures and Tables

**Table 1:** Comparative analysis of classification metrics across different machine learning algorithms.

Model	Accuracy	F1	Precision	Recall	ROC AUC	PR AUC
Decision Tree	0.7666 [0.7486–0.7847]	0.7860 [0.7668–0.8039]	0.7156 [0.6911–0.7409]	0.8719 [0.8503–0.8933]	0.8362 [0.8193–0.8533]	0.7675 [0.7380–0.7972]
Random Forest	0.7945 [0.7775–0.8111]	0.8209 [0.8036–0.8380]	0.7183 [0.6940–0.7434]	0.9580 [0.9435–0.9702]	0.8703 [0.8555–0.8848]	0.8319 [0.8052–0.8570]
XGBoost	0.7957 [0.7780–0.8116]	0.8203 [0.8031–0.8369]	0.7226 [0.6985–0.7478]	0.9488 [0.9339–0.9620]	0.8712 [0.8560–0.8867]	0.8346 [0.8083–0.8590]
CatBoost	0.8006 [0.7832–0.8172]	0.8198 [0.8024–0.8360]	0.7378 [0.7134–0.7623]	0.9224 [0.9048–0.9387]	0.8701 [0.8550–0.8853]	0.8327 [0.8082–0.8572]
LightGBM	0.7952 [0.7780–0.8121]	0.8159 [0.7976–0.8326]	0.7313 [0.7083–0.7569]	0.9228 [0.9059–0.9392]	0.8683 [0.8531–0.8832]	0.8309 [0.8043–0.8554]

## Data Availability

The data used in this study are sourced from the MIMIC-IV database, which is publicly available on PhysioNet (https://doi.org/10.13026/6mm1-ek67). Access to the database is restricted to credentialed users who have completed the CITI ‘Data or Specimens Only Research’ training and signed the Data Use Agreement.

## Abbreviations

The following abbreviations are used in this manuscript:

AP	Average Precision
AUC	Area Under the Curve
AUROC	Area Under the Receiver Operating Characteristic Curve
BMI	Body Mass Index
CatBoost	Categorical Boosting
CDSS	Clinical Decision Support System
CI	Confidence Interval
COPD	Chronic Obstructive Pulmonary Disease
EHR	Electronic Health Record
HFRS	Hospital Frailty Risk Score
HPA axis	Hypothalamic–Pituitary–Adrenal axis
ICD-10	International Classification of Diseases, 10th Revision
ICU	Intensive Care Unit
IL-6	Interleukin-6
LightGBM	Light Gradient Boosting Machine
MIMIC-IV	Medical Information Mart for Intensive Care IV
ML	Machine Learning
NHANES	National Health and Nutrition Examination Survey
OSA	Obstructive Sleep Apnea
pH	Potential of Hydrogen
pO_2_	Partial Pressure of Oxygen
PPV	Positive Predictive Value
PR AUC	Precision–Recall Area Under the Curve
REM	Rapid Eye Movement
ROC	Receiver Operating Characteristic
SHAP	SHapley Additive exPlanations
SpO_2_	Peripheral Oxygen Saturation
TNF-α	Tumor Necrosis Factor-alpha
XAI	Explainable Artificial Intelligence
XGBoost	Extreme Gradient Boosting

## Appendix A

**Table A1:** Summary of Hyperparameter Search Spaces and Best Values Obtained via Grid Search.

Model	Hyperparameter	Search Space (Grid)	Best Value
XGBoost	learning_rate	[0.01, 0.05, 0.1, 0.2]	0.01
	max_depth	[3, 5, 7, 10]	3
	n_estimators	[100, 200, 500]	500
	subsample	[0.6, 0.8, 1.0]	0.8
	colsample_bytree	[0.6, 0.8, 1.0]	0.6
	min_child_weight	[1, 3, 5]	5
	gamma	[0, 0.1, 0.2]	0.1
	n_estimators	[50, 100, 200]	200
	max_depth	[None, 10, 20, 30]	10
	min_samples_split	[2, 5, 10]	5
	min_samples_leaf	[1, 2, 4]	4
	max_features	[‘sqrt’, ‘log2’]	log2’
	bootstrap	[True, False]	TRUE
Random Forest	learning_rate	[0.05, 0.1, 0.2]	0.05
	max_depth	[−1, 5, 7, 10]	5
	n_estimators	[100, 200]	100
	num_leaves	[31, 50, 80]	80
	min_child_samples	[20, 50, 100]	50
	subsample	[0.6, 0.8]	0.8
	colsample_bytree	[0.6, 0.8]	0.8
	iterations	[100, 250]	250
	learning_rate	[0.05, 0.1]	0.05
	depth	[4, 6, 8]	4
	l2_leaf_reg	[1, 3, 5]	1
LightGBM	border_count	[32, 64]	64
	grow_policy	[‘SymmetricTree’, ‘Depthwise’]	SymmetricTree’
	max_depth	[None, 10, 20, 30]	10
	min_samples_split	[2, 5, 10]	5
	min_samples_leaf	[1, 2, 4]	4
	max_features	[None, ‘sqrt’, ‘log2’]	None
	criterion	[‘gini’, ‘entropy’]	entropy’
